# Development of an adolescent advisory group to inform sexual and reproductive health research for first- and second-generation immigrant adolescents in Canada: A community-based participatory action research study

**DOI:** 10.3389/frph.2022.930314

**Published:** 2022-10-28

**Authors:** Krooti Vyas, Samantha Louie-Poon, Salima Meherali

**Affiliations:** Edmonton Clinic Health Academy, Faculty of Nursing, University of Alberta, Edmonton, AB, Canada

**Keywords:** immigrant adolescent advisory group, community-based participatory action research, sexual and reproductive health, patient engagement evaluation tool, patient engagement in research

## Abstract

**Background:**

Despite the growing evidence supporting the benefit of engaging adolescents in research, the active engagement of immigrant adolescents in research is limited. Further, when exploring the sexual and reproductive health (SRH) needs of immigrant adolescents, utilization of adolescent advisory groups is finite. This study aimed to train and evaluate engagement of an adolescent advisory group (AAG) to inform SRH needs of immigrant adolescents in Canada.

**Methods:**

Using purposive sampling, 13 AAG members were recruited into this study. Members were trained in content related to SRH needs of adolescents and various research methodologies such as conducting a scoping review and qualitative interviews with adolescent participants. After 10 months of member engagement, their experiences were evaluated to identify areas of success and areas for improvement. These data were collected using the Public and Patient Engagement Evaluation Tool, which consisted of a Likert survey and open-ended questions, and analyzed in accordance to the Patient Engagement in Research (PEIR) framework.

**Findings:**

Ten members completed the evaluation survey. Likert survey responses were primarily positive. Majority of members showed positive demonstrations regarding various components of the PEIR framework, including contributions, support, research environment, and feeling valued.

**Conclusion:**

Findings illustrated that immigrant AAGs are constructive to informing SRH research. Not only can research teams benefit, but members are also empowered. This study provided the foundation for future immigrant adolescent engagement in research and knowledge translation, and effective means of evaluating engagement by utilizing the PEIR framework.

## Introduction

Adolescence refers to a transitional period of life between childhood and adulthood (1). According to the World Health Organization (WHO), adolescents are persons between the ages of 10 years and 19 years (1). Adolescents markedly experience intense changes in their mental, physical, and psychosocial growth, making this period a particularly sensitive and impressionable phase of life (1). Specifically, adolescents may face health challenges in relation to mental health (2, 3), substance abuse (4, 5), and sexual and reproductive health (SRH) (6, 7).

Despite this wide range of challenges, there remain very limited services and educational programs targeted towards SRH challenges in this age group (6). There are a wide breadth of problems intersecting and underpinning the reduced access to SRH services and educational programs, and result in low levels of SRH knowledge among adolescents (8). Research suggests that a low SRH knowledge base is linked with adolescents being less likely to access SRH resources and services (9, 10). Additionally, adolescents have low confidence in SRH services and possess feelings of shyness due to perceived negative connotations surrounding the concept of sex (9, 10). Fear of being criticized or embarrassed by friends, a concern that services are not fully confidential, parental reactions, and fear of judgment by staff are additional barriers that hinder youth from accessing these services (9, 10). Ultimately, the issues of availability and accessibility of SRH services and educational programs have substantial consequences for the health and well-being of youth populations. These consequences include, but are not limited to, teenage pregnancy, increased rates of morbidity and mortality, increased likelihood of contracting sexually transmitted infections, and partaking in risky sexual behaviour (11).

Interestingly, these consequences may be magnified for immigrant young people. Studies have shown that immigrant youth often identify closely with their parents' approval. Caal and colleagues found that parents of young Latina females played a crucial role in their SRH-seeking behaviours (12). In cases where immigrant parents disapprove of SRH services, adolescents consequently become discouraged to engage in help-seeking behaviours. In many immigrant cultures, the concepts of virginity and purity are emphasized. There is often stigma associated with the notion that having sex equates to impurity, which then serves as a hindrance to accessing services (12). Additionally, Shariati and colleagues studied SRH barriers for Iranian adolescent girls (13). They identified social and cultural barriers to accessing SRH services, including a fear of being seen as “loose”, concern of how society may view them, and holding the belief that seeking sexual and reproductive help does not align with their religion. With this knowledge, it is imperative to involve adolescents in SRH research as a critical next step in order to address and learn more about their unique needs.

### Benefits and challenges to adolescent engagement in research

In recent times, there has been an apparent shift from conducting research about youth and adolescents to directly involving them as collaborators of research (14–16). This shift to viewing adolescents as active participants in research is important as it allows for a more comprehensive understanding of the challenges they face, thus, allowing researchers and policymakers to better support them. Additionally, direct involvement gives adolescents a voice and the ability to incorporate ideas that may not have been accounted for in previous research (14–16).

However, there are noticeable practical and conceptual challenges to fully immersing this population in the research process, including the concept of tokenism (14). Young people are seen as symbols of an underrepresented population, and supposedly do not add much more value to research beyond that (14). Further, engaging with immigrant adolescents may have even deeper roots in tokenism. Because immigrant youth may be even less likely to participate in research, particularly in research centering on controversial topics such as SRH, they may experience tokenism to a greater extent. The lack of participation could be due to various reasons including hesitancy around the research topic, the research simply fails to account for them correctly, among others; however, the literature is still unclear (17). Further, tokenism may serve to disempower youth and discourage them from engaging in meaningful research, resulting in inaccurate findings that do not align with the needs of the youth (14).

### Immigrant adolescent engagement in SRH research

The immigrant youth population has been steadily increasing, and this population represents a significant proportion of immigrants to Canada (18). Despite the recognition of adolescence as an important developmental period, research on the health care needs, particularly SRH needs, of immigrant adolescents in the country is scarce. Because of the multitude of unique challenges faced by this population, such as adapting religious and cultural values to a new and unfamiliar environment, it is imperative that their perspectives are accounted for in research. Particularly, developing the neglected area of SRH research would encourage a more holistic understanding of immigrant youth needs and allow for more effective and accessible strategies to be implemented.

Despite the challenges identified earlier, attempts to incorporate immigrant adolescents as full research collaborators have significant positive implications. Extending beyond the notion of gaining a more holistic understanding of this population, allowing youth to be active and full participants in the research process is another way of empowering them (14–16). Stronger collaboration between researchers and adolescents is beneficial to both parties involved, because both are able to achieve their individual goals and purposes. Researchers are able to deliver more accurate research outcomes, which may progress to have impactful change in future SRH policies. These policies may actively reduce the consequences faced by immigrant adolescents regarding their individual and collective SRH. As well, the sense of empowerment – or the strength and conviction to control one's own life – that will be instilled in young immigrants will potentially give them the confidence they need to comfortably access SRH services and programs, no longer shy away from SRH topics, and support one another in their SRH journeys (14–16).

### Study purpose and objectives

This study adopted immigrant youth as collaborators of SRH research, rather than just participants, as a means of bridging this gap and to develop deeper insight into their needs. The aim was to allow immigrant adolescents to engage in SRH research and to explore their experiences of engagement in this research. The objectives were:

(a) to engage immigrant adolescents actively to provide advice, guidance, and knowledge that will inform various sexual and reproductive health research activities,
(b) to explore facilitators and challenges to immigrant adolescent engagement in SRH research, and
(c) to explore immigrant adolescents' perspectives on the value of their involvement in research.

## Methods

This study is a subsection of a larger community-based study exploring the sexual and reproductive needs of immigrant adolescents. The larger study was divided into four stages: (1) recruiting and training the adolescent advisory group (AAG); (2) conducting a scoping review on SRH needs and their impact on the holistic well-being of immigrant adolescents in Canada; (3) interviews with immigrant adolescents and parents on the influence of SRH needs on immigrant adolescents; and (4) an adolescent engagement evaluation. This paper is reporting on stages 1 and 4.

### Design

A community-based participatory action research (CBPAR) design was used to inform this study. Peer-based research models, such as CBPAR, provide sensitive and culturally appropriate inroads to “hard to reach” communities (19, 20). Knowing this, the authors adopted this design as a means of eliciting active engagement with the AAG.

### Participants and sample

The eligibility criteria for potential AAG participants included the following: (a) born outside of Canada and/or having a parent born outside of Canada, (b) between the ages of 14–19 years old, and (c) expressing interest in participation. The criteria were adapted as needed; if the authors deemed that an individual's insights may prove valuable even though they did not meet one aspect of the criteria, they were still qualified to participate on the discretion of the principal investigator (SM). AAG participants were recruited from various immigrant backgrounds, including Pakistan, Bangladesh, and India. Some participants born in Canada were included given that their parents were immigrants. Participants were recruited initially by a means of purposive sampling through personal affiliation with the research team. A snowball sampling approach was utilized when participants introduced interested individuals that they knew to the research team. The research team relied on this snowball sampling as a means of introducing broader ethnic diversities into the study; however, the study sample remained predominantly South Asian.

### Phase 1 data collection and management

At the time of recruitment, the authors discussed the expectations of engagement and the overall study by covering the following six key points. First, the team explained that adolescent participation was sought to ensure this project addressed the challenges faced by immigrant adolescents and the accessibility of SRH information and services to them. Secondly, adolescents were informed that they will be supported and their lived experience and perspectives are valued. Thirdly, adolescents will be engaging in shared leadership with the researchers to determine the strategies that will help to improve SRH informational needs and access to SRH services. Next, the AAG was advised that although individual input is desired, participation will also involve interacting with other adolescents. Then, the group was informed that diversity amongst members is expected and considered beneficial as the goal is to advocate for the needs of the larger immigrant community as a whole. Finally, the concepts of respect, trust, legitimacy, fairness, competence, and accountability as fundamental goals for the engagement processes were openly discussed. During this process, a confidentiality agreement form was circulated whereby participants signed their intention to ensure research information and data would remain private. In this form, their voluntary agreement and consent to participate were also obtained *via* a written record. Expected activities and commitment of the AAG were also discussed. The time commitment was based on the previous experience of the research team members, and was anticipated to be one-hour long virtual meetings every 2 months for an approximate period of 10 months; six training sessions were conducted. The timeline was flexible and adapted based on the progress and needs of the AAG. The first two authors prepared the training content, in consultation with the third senior author, and facilitated the training sessions. Members were permitted to revoke their consent to participate at any time. To foster meaningful engagement, AAG members received training on topics related to qualitative and quantitative methodologies, research ethics, and qualitative interview development. Other sessions that were conducted included an orientation to the study, a presentation of the interview findings from the larger study, and a final engagement evaluation. During orientation, members also completed a demographics information form.

### Phase 4 data collection and management

All adolescents who participated in the AAG were invited to complete the evaluation phase. The data from the engagement evaluation were collected using the Public and Patient Engagement Evaluation Tool (PPEET) (Appendix A) (21). A Research Assistant (RA) who was not a part of the study collected the data to prevent bias, given that the evaluation included an assessment of the interaction with the research team. Responses to the Likert survey were completed and submitted directly to the RA, who then sent anonymized responses for analysis. In addition, responses to the open-ended questions were collected through a focus group discussion and one individual meeting with a participant. The RA audio recorded both virtual meetings, which was transcribed verbatim and analyzed. The RA also informed the AAG members of their right to voice criticisms and on the importance of honest feedback in the evaluation. All data were anonymized prior to transcription and analysis to ensure honest feedback was provided.

### Phase 4 data analysis

The demographics form and the quantitative findings from the Public and Patient Engagement Evaluation Tool were analyzed using descriptive statistics. Once all participant demographics forms were collected, the data from each question was summed and analyzed against the total number of responses received. The qualitative data were analyzed using content analysis and using the Patient Engagement in Research (PEIR) framework (Appendix B) as a guide. The PEIR framework is a validated tool developed by Hamilton and colleagues that examines research engagement through the following themes: procedural requirements, benefits, contribution, convenience, feeling valued, research environment, support, and team interaction (22). Although formal codes were not generated, data were grouped and analyzed using the PEIR framework motifs based on the judgment and agreement of all authors. Common themes were then identified and reviewed within each motif.

### Ethics

Ethics approval was obtained from University of Alberta Ethics review board (Pro00097730).

## Findings

### Member information

Initially, eight adolescent members join the AAG. These members were all immigrant females from South Asian countries (Table 1). After the first meeting, the AAG expanded to include three more adolescents (10 female, 1 male). As the group grew, seven more recruits were added, three recruits were removed due to failure to contact, and two others were removed due to personal requests. The AAG was finalized with 13 members (10 female, 3 male) with the oldest participant being 23 years of age and the youngest being 16 years of age. Of the 13 members, 10 members participated in the AAG evaluation process. Majority (*n* = 7; 70%) of these participants were female; 50% (*n* = 5) were born in Canada, while 50% (*n* = 5) were born in Pakistan, Bangladesh, and India. All of these individuals' parents were immigrants. Majority (*n* = 7; 70%) of participants were completing post-secondary education.

**Table 1:** Demographic characteristics of individuals who participated in the adolescent advisory group (*N* = 10).

Variable	*N*	%
**Age**		
16 years old	2	20.0%
17 years old	1	10.0%
18 years old	–	–
19 years old	4	40.0%
19 + years old	3	30.0%
**Gender**		
Male	3	30.0%
Female	7	70.0%
**Languages spoken at home**		
English	10	45.5%
Bangla	1	4.5%
Arabic	1	4.5%
Gujarati	2	9.1%
Urdu	2	9.1%
Hindi	2	9.1%
Punjabi	4	18.2%
**Birthplace**		
Canada	5	50.0%
Pakistan	2	20.0%
Bangladesh	1	10.0%
India	2	20.0%
**Length of stay in Canada**		
Lived in Canada <1 year	–	–
Lived in Canada for 1–3 years	–	–
Lived in Canada for 4–9 years	–	–
Lived in Canada for 10 + years	2	20.0%
Lived in Canada all or most of their life	8	80.0%
**Level of education**		
Grade 9	–	–
Grade 10	–	–
Grade 11	–	–
Grade 12	3	30.0%
Post-secondary	7	70.0%
**Mother's birthplace**		
Canada	–	–
Egypt	1	10.0%
Pakistan	1	10.0%
India	6	60.0%
Bangladesh	1	10.0%
Outside of Canada (not specified)	1	10.0%
**Father's birthplace**		
Canada	–	–
Syria	1	10.0%
Pakistan	1	10.0%
India	6	60.0%
Bangladesh	1	10.0%
Outside of Canada (not specified)	1	10.0%
**Mother's highest level of education**		
Less than high school	–	–
High school	1	10.0%
College	1	10.0%
University	7	70.0%
Did not know	1	10.0%
**Father's highest level of education**		
Less than high school	–	–
High school	1	10.0%
College	2	20.0%
University	6	60.0%
Did not know	1	10.0%
**Other caregiver's level of education**		
Less than high school	–	–
High school	–	–
College	–	–
University	1	10.0%
Did not know	1	10.0%
Not applicable	8	80.0%

### Meeting design

**Figure 1:**
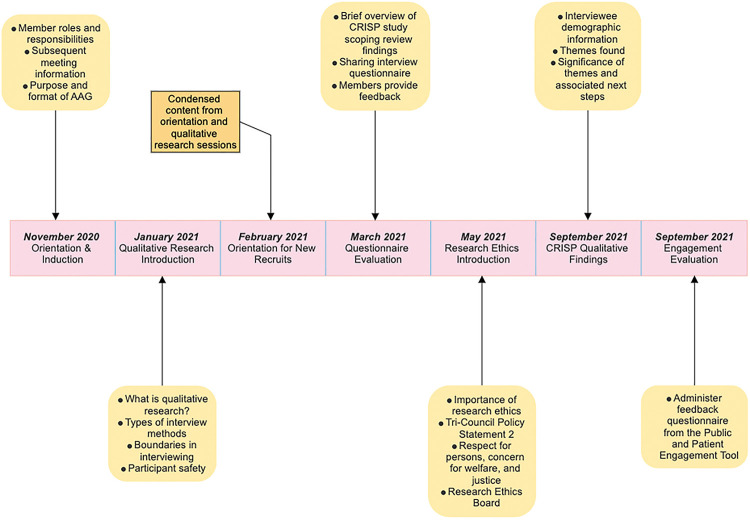
Outline of meetings and training sessions delivered to the AAG.

The first meeting acted as an orientation and outlined the format and purpose of the AAG, member roles and responsibilities, and the topic of subsequent meetings (Figure 1). Respectively, the topics of meetings to follow included an introduction to qualitative research, qualitative interview questionnaire development, an introduction to research ethics, communicating findings from the qualitative study interviews (e.g., third phase of the larger study), and feedback and AAG engagement evaluation. Each meeting consisted of an “ice breaker” and group activity to allow members to consolidate their learning and interact with one another.

### Engagement evaluation

#### Survey findings

For seven of the 14 items, participants agreed or strongly agreed with the statement (Table 2). Several items illustrated a conviction towards strong agreement: I was able to express my views freely; I feel that my views were heard; I feel that the input provided through this activity will be considered by the organizers; I think this activity will make a difference. For the other 7 items, a few participants remained neutral along with those that agreed or strongly agreed. No members disagreed or strongly disagreed with any of the statements.

**Table 2:** Results from the 14-item 5-point Likert survey of adolescent advisory group engagement evaluation (*N* = 10**).**

14 Items	Strongly Agree, *N* (%)	Agree, *N* (%)	Neither Agree nor Disagree, *N* (%)	Disagree, *N* (%)	Strongly Disagree, *N* (%)
1. The purpose of the activity was clearly explained.	7 (70%)	1 (10%)	2 (20%)	–	–
2. The supports I needed to participate were available.	7 (70%)	2 (20%)	1 (10%)	–	–
3. I had enough information to contribute to the topic being discussed.	4 (40%)	5 (50%)	1 (10%)	–	–
4. I was able to express my views freely	8 (80%)	2 (20%)	–	–	–
5. I feel that my views were heard.	8 (80%)	2 (20%)	–	–	–
6. A wide range of views on the topic were expressed.	3 (30%)	5 (50%)	2 (20%)	–	–
7. I feel that the input provided through this activity will be considered by the organizers.	9 (90%)	1 (10%)	–	–	–
8. The activity achieved its stated objectives.	7 (70%)	2 (20%)	1 (10%)	–	–
9. I understand how the input from this activity will be used.	6 (60%)	4 (40%)	–	–	–
10. I think this activity will make a difference.	8 (80%)	2 (20%)	–	–	–
11. As a result of my participation in this activity, I am better informed about challenges related to sexual and reproductive health.	6 (60%)	4 (40%)	–	–	–
12. As a result of my participation in this activity, I have greater trust in the research evidence.	5 (50%)	4 (40%)	1 (10%)	–	–
13. Overall, I am satisfied with this activity.	6 (60%)	4 (40%)	–	–	–
14. This activity was a good use of my time.	5 (50%)	4 (40%)	1 (10%)	–	–

Survey data were also analyzed based on the PEIR framework. In relation to the themes illustrated by the PEIR framework, components that were clearly met were convenience and support (The supports I needed to participate were available), feeling valued and team interaction (I feel that my views were heard; I feel that the input provided through this activity will be considered by the organizers), and research environment (I was able to express my views freely). Most members also elicited agreement with statements such as “I had enough information the contribute to the topic being discussed”, demonstrating a positive connection to the contribution component. Agreement was also noted for statements such as “As a result of my participation in this activity, I am better informed about challenges related to sexual and reproductive health” and “This activity was a good use of my time” highlighting the benefits element of the PEIR framework.

#### Focus group findings

Nine members participated in the focus group and 1 individual interview was conducted for a participant who was unable to attend the focus group. Narratives that were received from the focus group and individual interview were explored in relation to the themes of the PEIR framework. Although some responses may be applicable to more than one theme of the PEIR framework, we chose to attribute statements to the most applicable theme for simplicity and conciseness. Pseudonyms have been used for all participants.

##### Procedural requirements

With regard to improvements and future research directions, participants were keen on having more frequent meetings, as “it can be easy to forget what happened in the last meeting” (Aadhya, 19 years old). Some also mentioned wanting a secondary option of submitting input for those participants that may not feel comfortable sharing their thoughts in front of a larger audience. A participant suggested the idea of utilizing a Google Form as an alternative method.

##### Convenience

The convenience element was not explored in-depth by participants; however, an individual mentioned that he did not know if he “felt” that taking part in the study was convenient for him. When prompted and asked about what the barrier may have been, he expanded to state that he was “kinda lost on the information a little bit” (Ravi, 19 years old). This perspective may be an outlier, as the Likert survey showed that the majority of the individuals were satisfied with the study and their capacity to participate.

##### Contribution

Additionally, when members were asked about their overall experience, many expressed appreciation for hearing other members' stories. One member stated:

“… it's really comforting to know that, like, people like me, like, immigrants almost, um, go through the same thing and that, like, I’m not alone and we have basically, not the same experiences but the same kinds of experiences and we can, like, learn from that, and grow from it together” (Navaan, 19 years old).

Further, one notable theme that presented when members were encouraged to solicit suggestions for future research related to adolescent health was the impact of parental engagement in adolescence:

“… I think there should be more research done on how parents’ engagement in adolescents’ life affects their, uh, work in school …” (Lana, 22 years old).

“… something that’d be helpful would be, um, how immigrant parents with, um, that have adolescent children would deal with, like, mental health and stuff … because, like, for immigrants … I’d say, to them, it's not as, like, big of a deal” (Rani, 21 years old).

##### Support

Some members also demonstrated meaningful engagement by identifying ways in which researchers can further support individuals in adolescence. Members contributed factors in which additional research areas can be explored, particularly the impact that the virtual environment can have on adolescent health:

“… I was just thinking since like we’re like [unclear] age … [unclear] more research on cyberbullying and that effect on adolescent health [unclear] cyber bullying happens at a younger age” (Sonia, 16 years old).

“… I also think, like, how social media and everything affects, uh, kids and, like, teenagers and stuff, like, how much of it should be acceptable for a kid to see if, like, um, how seeing certain things affects children and adolescents” (Diya, 19 years old).

##### Team interaction

Moreover, participants were generally satisfied with their experience working with the AAG team through interactions with the research team and AAG members:

“… I think it was, like, pretty positive. I was able to interact with them. Like, easily.” (Ravi, 19 years old).

##### Research environment

Many members vocalized enjoying being able to fully express their different experiences and perspectives in a safe place:

“… I think the best thing was, uh, being able to express concern over adolescent, uh, specifically immigrant, sexual and reproductive health issues because we don’t really get to do it, um, in our own community because of a lot of like stigma and stuff. So, having a safe space to do it was very effective not only for us but also, um, the researchers” (Sonia, 16 years old).

##### Feeling valued

Members actively verbalized their feelings of being valued:

“I liked how a lot of the questions and things you did throughout the meetings were very open-ended. It allowed for a lot of different ideas to, like, to go through and really broaden people's perspectives on certain issues.” (Laksha, 21 years old).

When asked about potential feelings of being valued by team members and others, participants continued to express feelings of being valued:

“… Yeah, I would say so, ‘cause, like, they told us to give any input, if you, like, whenever you had an idea. And I felt like, they got all of us engaged. So, yeah.” (Ravi, 19 years old).

##### Benefits

Members identified tangential benefits of participation:

“… meeting new people and seeing what kind of experiences that we share [pause] that was the best part, to see that what we have in common and what we don’t.” (Vera, 19 years old).

In addition to the knowledge demonstrated about future adolescent health research, participants were able to identify potential ways the results of their participation will be used. AAG members introduced the ideas of SRH services tailored to immigrant adolescents and how the results will act as foundational for future research development:

**“**If I remember it's gonna be used to create an app for adolescents.” (Radha, 17 years old).

“… it's gonna be used to help, um, improve … the knowledge we already have on, like, adolescent sexual and reproductive health and, kind of, build upon what this research is already [unclear] to help out in these areas.” (Ash, 23 years old).

## Discussion

The findings of this study highlight that collaboration with young people on studies exploring immigrant adolescent SRH needs is critical. The findings also note that immigrant adolescents find value in expressing their views concerning this issue, and illustrated positive views on various aspects of the PEIR framework.

### Inclusive research environments

Participants actively emphasized the benefits of a safe and supportive research environment. Morris and colleagues identified and confirmed the importance of fostering a safe research environment (23). They highlighted that doing so encourages active cooperation from participants that are needed to achieve successful research outcomes. Promoting a healthy, working social relationship with participants is crucial to success. This notion is especially crucial when reporting on the cultural influences that impact immigrant adolescents and their views of SRH topics. Phase 3 of the larger community-based study exploring SRH needs of immigrant adolescents described findings that showcased the conservative perspectives of immigrant parents, which influenced how immigrant adolescents navigated – or refused to navigate – their SRH needs (24). Ultimately, these attitudes may exacerbate risk associated with poor SRH management. With this and the lack of a supportive community outside of the research environment in mind, it is imperative that immigrant adolescents find relief within a research community. Fortunately, continuing to facilitate positive research environments in SRH research will encourage participants to be more vocal about their perspectives and experiences, which, in turn, will develop more accurate findings and stimulate the development of appropriate and effective knowledge translation tools.

Further, positive team interactions are developed predominantly from having a supportive research environment (23). As this current study encouraged the importance of a healthy research environment, healthy team interactions came to fruition. As such, AAG members were able to contribute to research endeavors in a meaningful way. This study showed that an inclusive research environment promotes immigrant youths' satisfaction in contributing to research and having a space to freely talk. Havlicek and colleagues highlighted the relationship between contribution and the importance of an inclusive research environment for youth (25). The authors note that youth are more likely to open up about their experiences within a supportive environment. Therefore, future research engaging youth research partners should place emphasis on a positive and supportive research environment to ensure the meaningful contribution of adolescents.

### Youth empowerment and engagement

Arunkumar and colleagues identified the importance of lending adolescent advisory groups a voice to foster youth empowerment and facilitate accurate research outcomes (14). This study supports what has been stated in the literature. Within the context of our study, immigrant young people often feel their own communities silence SRH topics. Therefore, continuing to encourage their voice in SRH research in future investigations is deeply valuable.

The personal and future research and knowledge translation benefits vocalized by the AAG members shows the benefits of research engagement for participants and researchers alike. Although immigrant adolescents' engagement in SRH research has been severely lacking, participant comments align closely with the overall benefits of participatory research, specifically youth empowerment and improved research quality (26).

### Accommodations and practical improvements

There is currently little existing research focusing on the convenience element of involving immigrant adolescents in research. However, from the Likert survey employed in this study, majority of participants found that taking part in this study was convenient for them. Although not extensively explored, this may have been due to meetings being held at times that accounted for their busy schedules and that the meetings were held on a technological platform that the members were already familiar with. Future investigations will need to be conducted to explore this area particularly in light of existing pandemic measures.

Areas of improvement that were predominantly highlighted focused on procedural requirements. Specifically, they preferred more frequent meetings and a secondary option to provide input privately. Merves and colleagues facilitated a study that focused on engaging and sustaining youth in community-based participatory research (27). They structured their meetings to be held on a weekly basis and designed to focus on the short-term, rather than the larger long-term picture. The authors' findings illustrated that the youth participants particularly enjoyed these frequent meetings. With this knowledge and the findings from this current study, SRH research focusing on the needs of immigrant adolescents may benefit from more regularly scheduled sessions.

### Implications for research, education, and practice

The findings from our study add to the discussion on SRH needs by establishing that advisory groups, specifically those including immigrant adolescents, contribute to research development in a profound way. Most notably, they are able to provide valuable insight relating to questionnaire design, practical advice on successful implementation procedures that will increase the likelihood of reaching their demographic and encourage opportunities for future collaboration (28). Given that the body of literature on the involvement of immigrant adolescents as collaborators in research is quite lacking, this study acts as a pivotal, foundational piece of literature.

Unfortunately, regarding studies that have organized advisory groups, very few have utilized the advice and knowledge of its associated members (29). This notion illustrates a disparity with what the members of our study believed. Majority of the AAG members had indicated that they strongly agreed with the statement “I feel that the input provided through this activity will be considered by the organizers”. To create impact and change within a specific demographic group, it is imperative to utilize their advice and put it into action whenever possible; doing so encourages appropriate representation in subsequent policies and programs (30, 31). Our study implies that future investigations would need to focus on applying this finding in order to develop accessible knowledge translation tools.

Moreover, by encouraging a more direct, personal, and open line of communication with immigrant adolescents, the research team was able to learn of their relationship to research engagement more intimately. Our study was able to add to this small, but growing body of literature, particularly by ensuring a reflective attitude that aligns with the priorities of our target audience (31–33).

As well, studies have shown that many youth advisory group members often do not receive any formal training during, or prior to, establishment (16). This information is significant as it determines the extent to which participants can comfortably collaborate within the group and research coordinators and thus, impact the credibility of the findings. Our study attempted to begin bridging this gap and demonstrated the importance of appropriate training and facilitation, particularly noted by the positive engagement highlighted by the AAG members.

Based on the findings, continuing to add to this lacking area of research by conducting more studies examining the SRH needs of immigrant adolescents and including them in a collaborator capacity is crucial. These findings can be integrated into future research by continuing to foster an environment that celebrates the benefits of including immigrant adolescents as collaborators of research. Doing so encourages the expression of different perspectives and facilitates stimulating discussion that can later be applied in practice. The implementation of adolescent advisory group ideas is also encouraged. Researchers do not always personally identify with the demographic group in question (e.g., immigrant adolescents); therefore, their needs are not known intimately. For this reason, they may be able to identify opportunities for education and practical application that researchers may overlook (26).

### Limitations

This study establishes merit by placing emphasis on the inclusion of immigrant adolescents as collaborators in research; however, the AAG appeared to be primarily dominated by South Asian populations. Although the research team achieved a diverse range of viewpoints and engaging adolescents from various ethnic communities is an identifiable strength, it is possible that the voices of other immigrant backgrounds may not have been heard. Furthermore, the AAG emphasized perspectives from adolescents identifying as female, whereas viewpoints from participants identifying as male may not have been comprehensively explored. Therefore, the findings may not be generalizable to a larger population. Additionally, based on the focus group evaluation, it can be determined that some members may not have been comfortable expressing their perspectives on such a sensitive topic in front of an audience. As a result, it is possible that some important viewpoints may have been missed. Some participants were included into the study by means of affiliation with the research team and subjective deciding on the part of the principal investigator (SM). This limitation presents as a risk, due to the potential for the participants to inadvertently cooperate with the researchers' views despite their personal views. However, to minimize the bias the RA who collected Phase 4 data was not part of the research team and had no affiliation with any AAG member because the content pertains to interactions with the principal investigator and other members of the research team. In addition, data was anonymized before analysis to ensure honest feedback. In addition, the RA informed the AAG members of their right to voice criticisms and of the benefit of honest feedback in the evaluation before the focus group discussions.

## Conclusion

The lack of attention given to immigrant adolescent populations in a research collaborator role in existing research is a significant problem. Understanding adolescent perspectives regarding sensitive issues such as SRH is imperative to establishing appropriate and effective services and programs for this population. This study allowed for the generation of knowledge required to potentially advance this subsection of public health, and for adolescent advisory groups to be seen as an important factor that can provide significant contributions to further research endeavors and practical applications. Collaboration with the AAG will continue and they will be engaged in the development and design of innovative, context-specific knowledge translation strategies that will ultimately improve SRH outcomes of immigrant adolescents and their overall health and well-being. The experiences and reflections presented in this paper with the AAG will contribute to active immigrant adolescent engagement in SRH research.

## Data Availability

The raw data supporting the conclusions of this article will be made available by the authors, without undue reservation.

## Appendix A

### Public and Patient Engagement Evaluation Tool

**Instructions**

• We are interested in your feedback about the engagement activity that you recently participated in.
• The questionnaire is composed of several statements. Please indicate your level of agreement with each statement and check only one box for each statement.
• Please provide additional feedback in the comment boxes provided throughout the questionnaire.
• All information you provide will remain confidential.
• Thank you very much for your participation.

**Table:**

	Strongly Agree	Agree	Neither Agree nor Disagree	Disagree	Strongly Disagree
The purpose of the activity was clearly explained.					
The supports I needed to participate were available (e.g., travel, child care etc.).					
I had enough information to contribute to the topic being discussed.					
I was able to express my views freely.					
I feel that my views were heard.					
A wide range of views on the topic were expressed.					
I feel that the input provided through this activity will be considered by the organizers.					
The activity achieved its stated objectives.					
I understand how the input from this activity will be used.					
I think this activity will make a difference.					
As a result of my participation in this activity, I am better informed about challenges related to sexual and reproductive health.					
As a result of my participation in this activity, I have greater trust in the research evidence.					
Overall, I was satisfied with this activity.					
This activity was good use of my time.					

Open-ended questions

1. How do you think the results of your participation will be used?
2. What was the best thing about this engagement activity?
3. How satisfied are you with the frequency of tasks and activities related to this engagement activity?
4. What made you become interested in this engagement activity?
5. What was your overall experience with this engagement activity?
6. Please provide some suggestions for future research related to adolescent health.
7. Please identify at least one improvement we could make for future engagement activities.
